# Junior doctor daytime bleep audit

**DOI:** 10.1192/bjo.2021.239

**Published:** 2021-06-18

**Authors:** Asha Dhandapani, Sathyan Soundararajan, Rajvinder Sambhi

**Affiliations:** BCUHB

## Abstract

**Aims:**

There had been ongoing concerns with regard to covering daytime duty bleeps across the three sites in the Mental health Department, BCUHB, North Wales.

Frequent empty on-call slots meant some doctors being asked to hold the bleep between 9-5 in-order to cover the vacancy.

Some felt this added to the existing workload and that it was unfair and unsafe.

This issue was raised during a supervision session with the Educational supervisor, North Wales and an initial data collection was suggested.

**Method:**

Data were collected over 2 week period to look at the Daytime bleep duties between 9 am to 5 pm

We hoped the data would demonstrate certain patterns of the task being asked to perform.

**Result:**

The total number of bleeps were noted to be 249

Discharge notification and prescription writing was noted to be the commonest reason for bleep in East and Central while Routine review and Discharge notification was the reason to be bleeped major number of times in the West

Nearly 70% and 90% of the bleeps were found to be appropriate by the East and West respectively, while only a mere 15% were reported so in Central.

While 30% of these bleeps in the West were considered to be deferred, 70% bleeps were deferrable in the East and almost 95% in Central.

The general trend in all 3 centres was as follows:

All three centres have high numbers of bleeps for discharge, prescribing tasks and routine patient reviews

Most think planned discharge paperwork could be done in advance and jobs can be deferred if there is a ward/team doctor available

**Conclusion:**

A simple solution could be some jobs being planned ahead (e.g TTO/Discharge Summaries, Re-write charts) and done by the team/ward doctor. ECG could be arranged to be done by nurses/ECG technicians. Some nurses/HCAs are trained in phlebotomy, however, they have not been utilising the skills. That needed to be reinforced in safety huddles meeting.

Apart from these suggestions, we were also wondering about the impact of the service models and how the juniors placed in the community mental health unit could stay involved in their team inpatients

